# Commentary: Is the developmentally immature immune response in paediatric sepsis a recapitulation of immune tolerance?

**DOI:** 10.3389/fimmu.2019.02932

**Published:** 2019-12-19

**Authors:** Manoj Muthukuru

**Affiliations:** Health Sciences Center, University of Washington, Seattle, WA, United States

**Keywords:** immune system, endotoxins, Toll-like receptors, inflammation, neonatal sepsis, immune tolerance

## Introduction

This commentary is on the review article “*Is the developmentally immature immune response in paediatric sepsis a recapitulation of immune tolerance?”* by Maddux, A. B. and, Douglas, I. S., published in Immunology (2015) (1). In this review, the authors succinctly elaborate the immature state of the neonatal immune system and its innate immune responses, which are characteristically hypo-inflammatory. Contextually, these hypo-inflammatory responses of the neonatal immune system can be attributed to the low expression profiles of Toll-like Receptor (TLR4), the putative receptor that recognizes lipopolysaccharide (LPS) and also related to ineffective TLR signaling, which is eventually required for transcriptional activation and induction of pro-inflammatory cytokines (2). This hypo-inflammatory response is not only of the decreased production of pro-inflammatory cytokines but also of an exaggerated expression of anti-inflammatory mediators (1, 2).

In light of the neonatal immune response being hypo-inflammatory, however, we currently know very little as to why the prevalence, morbidity, and mortality of neonatal sepsis are significantly higher relative to pediatric sepsis affecting older children and adult sepsis (3–5). In an effort to address this apparent discrepancy with respect to bench-top findings vs. bedside outcomes, the contribution of immune or endotoxin tolerance in the neonatal immune system has been suggested (1). Since the immature neonatal immune system and the state of endotoxin tolerized immunoregulatory responses among adults are exemplified by the hypo-inflammatory response and present with striking similarities, such as low expression profiles of TLR4 and preponderance of anti-inflammatory cytokine signatures, we may arguably conclude that the overall characteristics of the neonatal immature immune responses differ from the mature immune responses in ways that are similar to the differences between endotoxin tolerance and endotoxin sensitized responses (1).

The complex molecular regulations that are associated with the induction of endotoxin tolerance are attempts to blunt the unfavorable aspects or the proverbial *double-edged sword* that connotes overall inflammatory responses (6); however, the downside of endotoxin tolerance also results in ineffective microbial killing and antigen presentation and appropriately inducing co-stimulatory signals for activating adaptive immune responses that could cause persistence of chronic inflammatory conditions or coexisting secondary infections during sepsis (7). These dampened adaptive immune responses, which are associated with endotoxin tolerance, present another dimension of confounding similarities with that of the neonatal immune system wherein, newborns not only have low amounts of circulating monocytes and dendritic cells but these cells also have lower levels of antigen-presenting MHC-II and co-stimulatory molecules, such as CD80 and CD86, compared to adults, which is indicative of the inability of the neonatal immune system to fully activate antigen-specific adaptive immune responses (2).

## Discussion

We must reiterate that the fundamental basis of endotoxin tolerance is exemplified by the resistance to subsequent LPS challenges after the host has been exposed to sub-lethal doses of LPS (8). Thereby, neonatal immune responses have to exhibit any of the aspects that are related to an endotoxin tolerized state, there must be prior exposure and sensitization to LPS. It is from this context that the overall immunoregulatory aspects that are associated with the induction of endotoxin tolerance are referred to as “*cellular reprogramming”* that defines the status of immune cells during sepsis or systemic inflammatory response syndrome (8). Computational biological methods and mathematical modeling have vigorously explored distinct network topologies that are associated with these distinct LPS exposures and the state of immune responsiveness through either LPS priming or endotoxin tolerance (9). Moreover, this state of cellular reprogramming that is associated with endotoxin tolerance is sustainable for extended period of time and therefore, these immunoregulatory aspects also demonstrate the attributes of innate memory (10, 11). In order for effectual tolerizing responses to partake in the overall immune responses, therefore, the ontology of the immune system should obtain instructions from the environmental signals that are predominantly derived from postnatal microbial colonization (12).

Moreover, at this juncture, we also observe that the molecular aspects of endotoxin tolerance have been extensively but exclusively investigated in adult leukocytes, either from humans or from animal models, and furthermore, these studies have established the mechanistic existence of the endotoxin tolerized states among adults after the clinical or experimental squeal of severe sepsis (8) and during localized chronic inflammatory conditions, such as periodontitis (13–15) and inflammatory bowel diseases (16). Recent investigations exploring the field of neonatal immunology have also critically analyzed sepsis and have associated immunoregulatory responses that are associated with immunosuppression with endotoxin tolerized states (17). However, we currently lack direct experimental evidence from clinical studies and relevant genomic analyses that can establish these distinctively altered immune responses during sepsis and associate these features with the age-related maturational states of the immune system (18, 19). Critical understanding of these immune and immunoregulatory functions through the prenatal, neonatal, and postnatal developmental stages have to be evaluated from the overall evolutionarily governing principles that are related to entropy; i.e., the metabolic programming during fetal development is tuned toward conservation of energy, and these aspects characteristically correlate with the immune responses that are resistant or homeostatic vs. responsive or tolerogenic (20, 21). These aspects have profound clinical implications as we routinely encounter altered physiological states during pediatric vaccinations when the host immune response is primed through vaccine adjuncts. However, from the overall health restorative aspects of the host, it is currently unclear how the immune tolerizing responses should be exploited.

Therefore, on the basis of the critical interpretation of the immunoregulatory aspects that are associated with the induction of endotoxin tolerance, along with the clinical presentations of neonatal and pediatric sepsis and the lack of established studies that have neither evaluated nor established the existence of this critical endotoxin tolerance response during the early stages of the development of the immune system, I present my hypothesis: Immature immune responses of the neonatal immune system are predominantly anti-inflammatory, but the mechanistic aspects of immune or endotoxin tolerance that share some of these immunologically similar signatures of hypo-inflammatory response must be distinctly different in the neonatal immune system that begins the transition from the sterile intrauterine environment into the world of microbial colonization and sensitization.

## Author Contributions

The author confirms being the sole contributor of this work and has approved it for publication.

### Conflict of Interest

The author declares that the research was conducted in the absence of any commercial or financial relationships that could be construed as a potential conflict of interest.

## Abbreviations

TLR: Toll-like receptor
LPS: lipopolysaccharide.
